# Dysmenorrhea in Polish Adolescent Girls: Impact on Physical, Mental, and Social Well-Being—Results from POLKA 18 Study

**DOI:** 10.3390/jcm13206286

**Published:** 2024-10-21

**Authors:** Michalina Drejza, Katarzyna Rylewicz, Ewa Majcherek, Joanna Barwińska, Grzegorz Łopiński, Małgorzata Mizgier, Katarzyna Plagens-Rotman, Magdalena Pisarska-Krawczyk, Grażyna Jarząbek-Bielecka, Witold Kędzia

**Affiliations:** 1 Cambridge University Hospitals NHS Foundation Trust, Cambridge CB2 0QQ, UK; 2 National Medical Institute of the Ministry of the Interior and Administration, 02-507 Warsaw, Poland; katerylewicz@gmail.com; 3 Faculty of Medicine, Poznan University of Medical Sciences, 61-701 Poznan, Poland; ewaa.majcherek@gmail.com; 4 Murcki Hospital, 40-749 Katowice, Poland; jbarwinska21@gmail.com; 5 Samodzielny Publiczny Zakład Opieki Zdrowotnej, 08-110 Siedlce, Poland; gregory.lopinski@gmail.com; 6 Department of Sports Dietetics, Faculty of Health Sciences, Poznan University of Physical Education, Królowej Jadwigi 27/39, 61-871 Poznan, Poland; mizgier@awf.poznan.pl; 7 Division of Gynaecology, Department of Gynaecology, Poznan University of Medical Sciences, 61-701 Poznan, Poland; plagens.rotman@gmail.com (K.P.-R.); graja@ump.edu.pl (G.J.-B.); witold.kedzia@poczta.fm (W.K.); 8 Department of Nursing, The President Stanislaw Wojciechowski Calisia University, 62-800 Kalisz, Poland; magmp@op.pl

**Keywords:** dysmenorrhoea, menstruation, adolescent gynaecology, adolescents, mental health, risk behaviours, violence, school health, Poland

## Abstract

**Background**: Dysmenorrhea, characterised by painful menstrual cramps, is a pressing issue among adolescent girls globally. It significantly impacts their quality of life and has been associated with increased mental health issues and engagement in risky behaviours like smoking. In Poland, there is limited research on menstrual health, emphasising the need for a study to understand dysmenorrhea experiences and their impact on young menstruating individuals. **Methods**: This research project investigated the effects of dysmenorrhea on quality of life and school attendance, as well as its associations with non-communicable diseases, including mental health among adolescent girls in Poland. Additionally, the study examined risk factors for non-communicable disease development, including high-risk health behaviours and exposure to violence. The study utilised a cross-sectional design, administering self-reported questionnaires in high schools and vocational schools in six voivodeships (regions) in Poland. The analysis was performed using the R language in the Rstudio environment. *p*-value < 0.05 was considered significant. **Results**: A significant percentage of respondents experienced heavy menstruation, irregularity, and pain. Adolescents with dysmenorrhea reported higher rates of school absenteeism, mental health issues (such as anxiety and panic attacks), and a higher likelihood of engagement in risk behaviours like smoking and illicit drug use. The study also identified associations between dysmenorrhea and experiences of violence, including sexual abuse and intimate partner violence, as well as links to self-harm and suicidal ideation. **Conclusions**: These findings contribute to understanding dysmenorrhea among Polish adolescent girls, emphasising the need for tailored interventions and support services. The study underscores the necessity of addressing menstrual health comprehensively, considering its impact on various aspects of young women’s lives and promoting their overall well-being.

## 1. Introduction

Menstruation is a natural and physiological process; however, for many, it is accompanied by menstrual pain, scientifically known as dysmenorrhea. Dysmenorrhea, defined as painful menstrual cramps of uterine origin, is a prevalent condition experienced by adolescent girls worldwide. It can affect up to 70% of women and girls, regardless of their country’s economic status. Primary dysmenorrhoea is considered menstrual pain without underlying pathology, and it typically initially occurs in adolescence. It is the most common cause of dysmenorrhoea in people below the age of 25. Secondary dysmenorrhoea is menstrual pain with an identifiable cause, most often endometriosis [1,2,3].

While menstrual pain is common [4], its impact on various aspects of adolescents’ lives remains an area of concern. The discomfort and inconvenience caused by menstrual pain can lead to a decreased overall quality of life, affecting physical, emotional, and social well-being. This condition not only causes physical discomfort but can also significantly impact various aspects of life, including increased mental health issues, and engagement in high-risk behaviours [5,6]. Furthermore, dysmenorrhea in adolescent girls has been linked to several non-communicable diseases such metabolic dysfunctions including those affected by lifestyle (e.g., diabetes, obesity, etc.), or autoimmune conditions (e.g., inflammatory bowel diseases, lupus, rheumatoid arthritis, etc.) [7].

Studies have shown a higher prevalence of depression, anxiety, and poor overall mental health among girls experiencing severe menstrual pain. The chronic nature of menstrual pain can exacerbate these mental health issues, leading to a significant decline in psychological well-being [8,9]. Furthermore, adolescents experiencing menstrual pain may be more likely to engage in high-risk behaviours as a coping mechanism, including self-harm and smoking cigarettes [10]. Additionally, adolescent girls may turn to smoking to alleviate the symptoms of dysmenorrhea [11]. However, there was no association between alcohol and dysmenorrhoea in recent studies [12,13].

Dysmenorrhea also affects the school performance of adolescent girls and other menstruators. The pain and discomfort associated with dysmenorrhea often lead to increased school absenteeism and lower activity levels—including lower physical education classes attendance, resulting in missed educational opportunities and potential setbacks academically [14].

Despite the far-reaching impact of dysmenorrhea on the lives of adolescent girls, there is a significant gap in population-level research around menstrual health in Poland. Thus, it is imperative to conduct a study that specifically focuses on surveying Polish girls, providing insights into their experiences with menstrual pain. This study intends to contribute to the existing body of knowledge on menstrual health, shed light on the unique challenges faced by Polish girls, and inform the development of tailored interventions and support services.

## 2. Materials and Methods

This research is part of the POLKA 18 initiative, a cross-sectional study led by youth, aimed at evaluating the knowledge, attitudes, and behaviours of Polish adolescents on health-related topics, particularly focusing on sexual and reproductive health and menstrual health. To circumvent restrictions on researching these sensitive topics in Polish schools, an inclusive interdisciplinary logic model was developed to address various aspects of adolescent health, first trialed during youth consultations at an educational institution in December 2018.

The study consisted of two phases: an initial pilot study (Phase I) and a subsequent main study (Phase II), both employing self-administered paper questionnaires. During Phase I, questionnaires were distributed in five regions (voivodeships) of Poland: Mazowieckie (central Poland), Wielkopolskie (west of Poland), Lubelskie (east of Poland), Śląskie (south of Poland) and Pomorskie (north of Poland). Following Phase I, the research group held a meeting to evaluate any issues encountered and plan further questionnaire distribution. In Phase II, a sixth polish region was included—Zachodniopomorskie (north-west Poland)—to ensure geographical representation.

Medical students associated with IFMSA-Poland distributed these questionnaires in high schools and vocational schools, with consent from both the schools and the students being mandatory. The selection of schools was based on past collaborations or engagement with IFMSA-Poland, ensuring the participation of final-year high school students and penultimate-year vocational school students above 18 years old and not requiring parental consent. Questionnaires were completed during health-related educational sessions overseen by IFMSA-Poland representatives, where students were briefed on the study, including the topic, their rights, anonymity, and the non-collection of personal data. Contact information for the research team was also provided to the participants. The questions from the questionnaires that were used in this study can be found in Appendix A.

Questionnaires were disseminated in six Polish voivodeships, with data from the Zachodniopomorskie region combined with data from the Pomorskie region to maintain statistical integrity. Data from the POLKA 18 survey were utilised to extract information on age, gender, region of residence, menstrual health, mental health, risk behaviours, school attendance, and exposure to violence. Prior to analysis, all data underwent careful scrutiny for accuracy and completeness. Statistical tests such as the Chi-square Pearson test and the Wilcoxon test were employed for intergroup comparisons of qualitative and non-normally distributed quantitative variables, respectively. Kendall’s coefficient was used to evaluate correlations between variables, with the data analysis conducted using the R programming language in the Rstudio environment, setting the significance at a *p*-value < 0.05.

All student participants provided voluntary informed consent, and the project obtained ethical clearance from the Ethics Committee of Poznan University of Medical Sciences. Data handling adhered to the ethical principles of the Declaration of Helsinki, ensuring anonymity throughout the study process.

## 3. Results

### 3.1. General Characteristics of the Study Sample

In total, 1545 completed questionnaires collected and filled in by menstruating individuals were included in the final analysis of the study. The general characteristics of the sample can be found in Table 1 below.

### 3.2. Characteristics of Menstrual Cycle

A total of 42% of respondents considered their menstruation to be heavy or very heavy. When asked about the regularity of menstruation, 21% revealed that it was irregular or very irregular. A total of 51% reported their menstruation as painful or very painful.

### 3.3. Impact on School Attendance

People who reported dysmenorrhea have significantly more often missed both physical education (*p* < 0.001) and overall school classes (*p* < 0.001) in comparison to people who have not reported painful periods.

**Figure 1:**
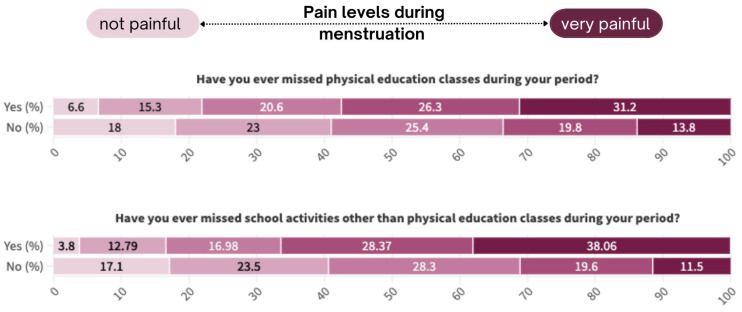
School attendance stratified by pain levels during menstruation.

A summary of these responses are shown in Figure 1 below.

### 3.4. Menstrual Pain and Risk Factors for Development of Non-Communicable Diseases

#### 3.4.1. Associations with High-Risk Health Behaviours

A positive association was found between the level of pain experienced during menstruation and ever having smoked a cigarette (*p* < 0.002). However, there was no clear association between dysmenorrhoea and the number of cigarettes smoked in the last 30 days (*p* = 0.684).

No association was found between dysmenorrhoea and the number of alcoholic drinks consumed in the last 30 days or ever having been drunk.

There was no significant association found between the level of pain experienced during menstruation and having ever tried marijuana. However, a positive association was found between dysmenorrhoea and having tried other illicit drugs (*p* = 0.013).

The findings are summarised in Table 2 below.

#### 3.4.2. Associations with Experience of Violence

There was significant association between experience of violence and dysmenorrhea. Adolescents who were forced to perform unwanted acts such as kissing, touching or having sexual intercourse reported dysmenorrhea more often than people without this experience (*p* = 0.001). This association was less significant with regard to experiencing violence for people they stayed in significant relationships with (*p* = 0.056).

**Figure 2:**
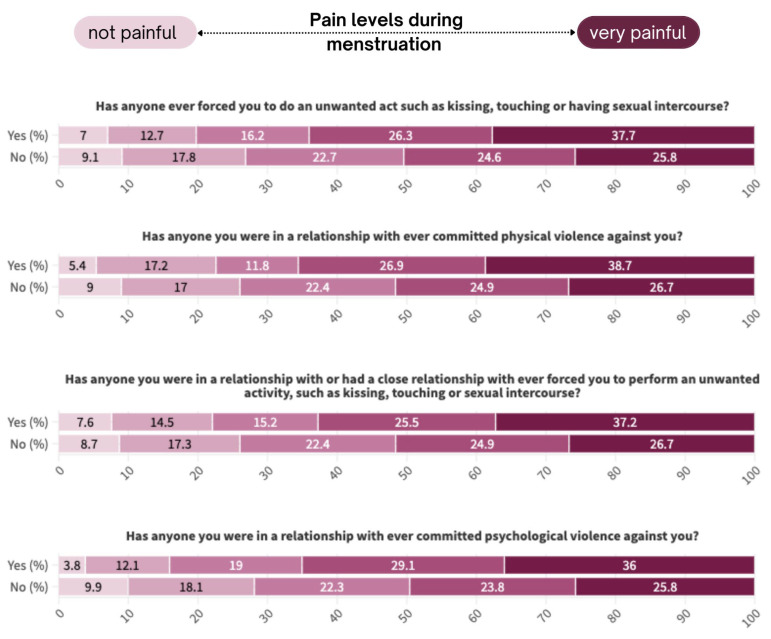
Experience of violence stratified by levels of pain during menstruation.

Respondents who experienced both physical (*p* = 0.034) and psychological (*p* = 0.001) intimate partner violence significantly more often reported a higher degree of painful periods. A summary of these responses are shown in Figure 2 below.

### 3.5. Menstrual Pain and Non-Communicable Diseases (NCDs)

#### 3.5.1. Associations with Mental Health Issues

Individuals who reported experiencing period pain were more likely to experience negative mental health issues in comparison to the individuals without pelvic pain.

There was an association between the experience of dysmenorrhoea and the occurrence of anxiety (*p* = 0.001) and panic attacks (*p* = 0.007).

Additionally, there was a strong association between period pain and self-harm behaviours (*p* = 0.011). People who marked their pain as heavy (4) and very heavy (5) were more likely to self-harm in comparison to those who marked their pain levels as lower (1–3).

This same observation can be made for individuals expressing suicidal ideations; they were more likely to report higher levels of period pain (*p* = 0.038). Menstruators who marked their pain as heavy (4) and very heavy (5) were more likely to have thought about suicide in comparison to those who marked their pain levels as lower (1–3).

There was also a positive association between the perceived level of stress and reported pain during menstruation with *p* < 0.001. A summary of these responses and their corresponding *p*-values are shown in Table 3 below.

#### 3.5.2. Associations with Other Non-Communicable Diseases (NCDs)

Other NCDs also exhibit prominently among individuals experiencing menstrual pains in comparison to the ones, who have not reported such problems. A strong correlation was observed between dysmenorrhea and the prevalence of allergies (*p* = 0.002) and other health complications (*p* = 0.004). Furthermore, hypertension (*p* = 0.029), heart and other vascular diseases (*p* = 0.022), gastrointestinal disorders (*p* = 0.04), and thyroid diseases (*p* = 0.019) were found to be more prevalent among individuals reporting pelvic pains.

However, no definitive correlation was found between dysmenorrhea and other conditions such as diabetes (*p* = 0.465), asthma (*p* = 0.419), psoriasis (*p* = 0.285), acne (*p* = 0.373), or musculoskeletal disorders (*p* = 0.172).

The associations are detailed in Table 4 below.

## 4. Discussion

### 4.1. Main Findings and Their Implications

The findings from our study provide valuable insights into the experiences and behaviours related to menstruation among the respondents. A significant proportion, 42%, considered their menstruation to be heavy or very heavy, while 21% reported irregular periods. More than half, 51%, reported experiencing painful or very painful periods. These findings are consistent with existing literature findings [3].

Furthermore, the impact of dysmenorrhea on individuals’ daily lives, including school life, was evident. Those who reported dysmenorrhea were more likely to miss physical education and more school classes overall compared to those without painful periods. This highlights the issue of school absenteeism among menstruating adolescents and the potential impact of poorly managed dysmenorrhoea on school performance. Studies have shown that severe menstrual pain can impair daily activities, such as academic performance, active participation in school life, and engaging in extracurricular activities. The reluctance to attend school due to fear of experiencing pain in public settings or the unavailability of adequate menstrual hygiene facilities can contribute to a vicious cycle of academic underachievement. This disruption can have long-term consequences for their educational attainment and future prospects [15,16,17]. It is essential for schools to create a supportive environment that acknowledges and accommodates the needs of students with menstrual health issues. By promoting open communication, providing access to pain management resources, and implementing flexible attendance policies, schools can help reduce absenteeism related to dysmenorrhea and support the well-being of all students.

The presence of negative predictors of mental health, such as self-harm and suicidal ideation, was associated with higher levels of period pain. Dysmenorrhea was also linked to anxiety, panic attacks, and increased stress levels. Studies have shown that the stigma surrounding menstruation in some societies, including Poland, can further contribute to feelings of shame and embarrassment, amplifying the psychological burden on affected individuals. The emotional toll of dysmenorrhea can have a profound impact on their daily functioning, social interactions, and self-esteem [8,9]. Our results show a need for mental health support in adolescents with menstrual pain, as well as effective management of organic health problems in adolescents with mental health problems.

Interestingly, there was a positive association between the level of pain experienced during menstruation and having ever smoked a cigarette. However, no clear association was found between dysmenorrhea and the number of cigarettes smoked in the last 30 days. There were no associations found between dysmenorrhea and alcohol consumption or marijuana use. However, a positive association was identified between dysmenorrhea and having tried other illicit drugs. Both smoking and alcohol intake pose immediate health risks and contribute to long-term detrimental effects on their overall well-being. The findings suggest the need to educate adolescents about risky behaviours and how they affect health and establish support programmes for those involved in them. The lack of adequate support systems and coping mechanisms exacerbates the susceptibility of adolescents to these harmful behaviours, highlighting the urgent need for comprehensive menstrual health education and access to healthcare services.

Experiences of violence, both forced sexual acts and intimate partner violence, were significantly associated with dysmenorrhea. Adolescents who reported being forced into unwanted sexual acts and those who experienced physical and psychological intimate partner violence had a higher prevalence of painful periods. A recent systematic review found a significant association between a history of sexual assault and overall gynaecologic morbidity, including the presence of dysmenorrhoea [18]. Additionally, another systematic review demonstrated that the number and severity of adverse childhood experiences, including sexual abuse, were associated with an increased risk of developing dysmenorrhoea [19]. It is crucial to address these aspects in the context of menstruation and provide support to individuals who have experienced such violence.

In terms of physical health, gastrointestinal, cardiovascular and thyroid problems, as well as allergies and other conditions not specifically characterised by us, have been observed to be more common in people with higher levels of menstrual pain. Autoimmune conditions can cause fatigue and inflammation, which may contribute to increased menstrual pain and discomfort during periods [7]. Establishing these correlations could prove beneficial in the treatment of adolescents in general practitioners’ offices or other internal medicine specialists. Giving greater attention to the issue of dysmenorrhea may be essential in ensuring overall physical well-being.

This study aims to explore the impact of dysmenorrhoea on quality of life, school absenteeism, non-communicable diseases, including mental health, and risk factors for their development: high-risk behaviours and exposure to violence among Polish adolescent girls. Ultimately, the findings will promote a better understanding of dysmenorrhea in the Polish context, paving the way for interventions that enhance the overall well-being and menstrual health of young women in Poland.

It is important to consider these findings when developing interventions and policies aimed at improving menstrual health and well-being. Further research is warranted to explore additional factors influencing menstruation experiences and develop comprehensive strategies that address the diverse needs of individuals regarding menstrual pain management and overall menstrual health.

### 4.2. Strengths and Limitations of the Study

The analysis utilised data from the POLKA 18 study, which has a large sample size representative of the Polish population. The study accounts for the geographical distribution and representation of different regions, enabling a thorough examination of the adolescent population in Poland. Nevertheless, the scope of our research was constrained by the legal age of consent, which is set at 18 years old in Poland. Consequently, we were unable to encompass adolescents within the broader definition, as our focus was solely on individuals aged 18 and above.

Convenience sampling was used in this study, which may have introduced some bias. The team faced challenges in reaching a wider student population as some schools refused to take part in the survey due to its coverage of sexual and reproductive health topics, which is a frequent problem in Poland. To address this problem, in some cases, we recruited schools to participate in our study based on previous cooperation.

The questionnaires were self-reported, enabling us to explore and obtain more candid responses on sensitive topics such as smoking, alcohol consumption, violence experience or menstrual health. However, the accuracy of the responses could not be verified, and external factors that may have influenced them could not be accounted for.

## 5. Conclusions

In summary, the findings of this study revealed that a considerable percentage of respondents experience heavy menstruation, irregularity, and pain. Adolescents with dysmenorrhea reported higher rates of school absenteeism, mental health issues (including anxiety and panic attacks), and an increased likelihood of engaging in risk behaviours like smoking and illicit drug use. The study also identified associations between dysmenorrhea and experiences of violence, including sexual abuse and intimate partner violence, as well as links to self-harm and suicidal ideation.

These findings contribute to understanding dysmenorrhea among Polish adolescent girls, emphasising the need for tailored interventions and support services. The study underscores the necessity of addressing menstrual health comprehensively, considering its impact on various aspects of young women’s lives and promoting their overall well-being.

In conclusion, the associations between menstrual pain and its multifaceted impacts on the lives of Polish adolescents are significant and far-reaching. Addressing the physical, psychological, and socio-cultural dimensions of menstrual health is essential for promoting the well-being and empowerment of adolescent girls in Poland. Comprehensive interventions that prioritise menstrual health education, access to menstrual hygiene products, and mental health support are imperative to mitigate the adverse effects of menstrual pain and foster a supportive environment for adolescent girls to thrive. Addressing the structural barriers to menstrual health education and access to menstrual hygiene products is crucial for promoting school attendance and retention among adolescent girls. By acknowledging and addressing these challenges, we can create a more equitable society where menstruation is no longer a barrier to the full realisation of adolescents’ rights and potential.

## Figures and Tables

**Table 1:** General characteristics of the sample (n = 1545) (%).

Age	
18	1216 (78.7%)
19	250 (16.2%)
Above 19	79 (5.1%)
Gender	
Female	1538 (99.5%)
Other	7 (0.5%)
Region of Poland	
Mazowieckie	414 (26.8%)
Śląskie	294 (19%)
Wielkopolskie	360 (23.3%)
Pomorskie and Zachodniopomorskie	292 (18.9%)
Lubelskie	184 (11.9%)
Residence	
City over 100,000	505 (32.8%)
City under 100,000	485 (31.5%)
Rural area	548 (35.6%)

**Table 2:** Engaging in high-risk behaviours stratified by levels of pain during menstruation.

n	Pain Levels during Menstruation (Scale 1–5)					*p*-Value
	1	2	3	4	5	
	132	256	327	378	420	
Have you ever smoked cigarettes? N (%)						
yes	82 (62.1)	150 (59.1)	208 (63.8)	258 (68.4)	305 (73.0)	0.002
no	50 (37.9)	104 (40.9)	118 (36.2)	119 (31.6)	113 (27.0)	
In the last 30 days, on how many days have you had a cigarette? N (%)						
0 days	80 (61.1)	166 (65.6)	188 (58.6)	223 (59.8)	236 (57.3)	0.684
1–2 days	15 (11.5)	26 (10.3)	44 (13.7)	41 (11.0)	48 (11.7)	
3–5 days	6 (4.6)	10 (4.0)	16 (5.0)	26 (7.0)	29 (7.0)	
6–9 days	7 (5.3)	11 (4.3)	10 (3.1)	15 (4.0)	14 (3.4)	
10–19 days	8 (6.1)	8 (3.2)	14 (4.4)	18 (4.8)	20 (4.9)	
20–29 days	5 (3.8)	4 (1.6)	17 (5.3)	12 (3.2)	13 (3.2)	
Everyday	10 (7.6)	28 (11.1)	32 (10.0)	38 (10.2)	52 (12.6)	
In the last 30 days, on how many days did you drink at least one alcoholic drink? N (%)						
0 days	27 (20.6)	58 (22.8)	78 (24.0)	68 (18.0)	73 (17.4)	0.231
1–2 days	45 (34.4)	80 (31.5)	100 (30.8)	130 (34.4)	126 (30.1)	
3–5 days	31 (23.7)	56 (22.0)	81 (24.9)	93 (24.6)	107 (25.5)	
6–9 days	19 (14.5)	36 (14.2)	35 (10.8)	51 (13.5)	76 (18.1)	
10–19 days	7 (5.3)	14 (5.5)	24 (7.4)	28 (7.4)	24 (5.7)	
20–29 days	0 (0.0)	4 (1.6)	6 (1.8)	7 (1.9)	8 (1.9)	
Everyday	2 (1.5)	6 (2.4)	1 (0.3)	1 (0.3)	5 (1.2)	
Have you ever been drunk? N (%)						
yes	95 (72.0)	185 (72.8)	250 (76.9)	295 (78.0)	338 (80.9)	0.086
no	37 (28.0)	69 (27.2)	75 (23.1)	83 (22.0)	80 (19.1)	
Have you ever tried marijuana? N (%)						
yes	50 (38.2)	83 (32.4)	106 (32.4)	147 (38.9)	157 (37.5)	0.260
no	81 (61.8)	173 (67.6)	221 (67.6)	231 (61.1)	262 (62.5)	
Have you ever tried drugs other than marijuana (e.g., LSD, cocaine, ecstasy, hashish, heroin)? N (%)						
yes	13 (9.9)	21 (8.2)	13 (4.0)	38 (10.1)	45 (10.8)	0.013
no	118 (90.1)	235 (91.8)	314 (96.0)	340 (89.9)	373 (89.2)	

**Table 3:** Mental health predictors stratified by pain levels during menstruation.

n	Pain Levels during Menstruation (Scale 1–5)					*p*-Value
	1	2	3	4	5	
	132	256	327	378	420	
In the past year, have you ever had a panic attack? N (%)						
Yes	49 (37.1)	99 (38.8)	151 (46.6)	189 (50.3)	206 (49.4)	0.007
No	83 (62,9)	156 (61.2)	173 (53.4)	187 (49.7)	211 (50.6)	
In the past year, have you ever felt anxious? N (%)						
Yes	67 (50.8)	124 (48.8)	200 (61.3)	230 (61.5)	262 (62.8)	0.001
No	65 (49.2)	130 (51.2)	126 (38.7)	144 (38.5)	155 (37.2)	
Have you thought about suicide in the past year? N (%)						
Yes	24 (18.3)	45 (17.6)	66 (20.4)	93 (24.8)	109 (26.3)	0.038
No	107 (81.7)	210 (82.4)	257 (79.6)	282 (75.2)	305 (73.7)	
In the past year, have you self-harmed yourself? N (%)						
Yes	8 (6.1)	25 (9.8)	22 (6.8)	51 (13.6)	52 (12.5)	0.011
No	123 (93.9)	230 (90.2)	302 (93.2)	325 (86.4)	365 (87.5)	
On a scale of 1 (very low) to 10 (very high), how would you rate your stress level? N (%)						
1	3 (2.3)	5 (2.0)	1 (0.3)	0 (0.0)	9 (2.2)	<0.001
2	8 (6.2)	11 (4.3)	8 (2.5)	8 (2.2)	11 (2.6)	
3	14 (10.8)	20 (7.9)	15 (4.7)	26 (7.0)	17 (4.1)	
4	13 (10.0)	35 (13.8)	31 (9.6)	37 (10.0)	24 (5.8)	
5	24 (18.5)	43 (17.0)	47 (14.6)	42 (11.3)	53 (12.7)	
6	13 (10.0)	30 (11.9)	36 (1.2)	41 (11.1)	33 (7.9)	
7	12 (9.2)	37 (14.6)	53 (16.5)	56 (15.1)	49 (11.8)	
8	18 (13.8)	34 (13.4)	69 (21.4)	81 (21.8)	83 (20.0)	
9	8 (6.2)	23 (9.1)	21 (6.5)	36 (9.7)	65 (15.6)	
10	17 (13.1)	15 (5.9)	41 (12.7)	44 (11.9)	72 (17.3)	

**Table 4:** Presence of non-communicable diseases stratified by levels of menstrual pain.

n	Pain Levels during Menstruation (Scale 1–5)					Total	*p*-Value
	1	2	3	4	5		
	132	256	327	378	420	1513	
Diabetes							
Yes	1 (4.2)	3 (12.5)	3 (12.5)	9 (37.5)	8 (33.3)	26	0.465
No	129 (8.8)	250 (17.1)	317 (21.6)	363 (24.8)	406 (27.7)	1493	
Hypertension							
Yes	6 (12.5)	9 (18.8)	4 (8.3)	8 (16.7)	21 (43.8)	49	0.029
No	124 (8.6)	245 (17.0)	316 (21.9)	364 (25.2)	393 (27.3)	1471	
Heart and other vascular diseases							
Yes	4 (3.4)	16 (13.8)	23 (19.8)	27 (23.3)	46 (39.7)	119	0.022
No	126 (9.2)	237 (17.3)	297 (21.6)	345 (25.1)	368 (26.8)	1400	
Thyroid diseases							
Yes	10 (6.7)	17 (11.3)	24 (16.0)	47 (31.3)	52 (34.7)	154	0.019
No	120 (9.0)	236 (17.6)	296 (22.1)	325 (24.3)	362 (27.0)	1365	
Gastrointestinal diseases							
Yes	5 (4.5)	10 (9.1)	29 (26.4)	28 (25.5)	38 (34.5)	111	0.04
No	125 (9.1)	243 (17.6)	291 (21.1)	344 (24.9)	376 (27.3)	1408	
Asthma							
Yes	9 (7.2)	16 (12.8)	26 (20.8)	31 (24.8)	43 (34.4)	130	0.419
No	121 (8.9)	236 (17.3)	294 (21.6)	342 (25.1)	371 (27.2)	1389	
Allergies							
Yes	25 (6.1)	61 (14.9)	74 (18.1)	113 (27.6)	136 (33.3)	417	0.002
No	105 (9.7)	192 (17.7)	247 (22.8)	259 (23.9)	279 (25.8)	1104	
Psoriasis							
Yes	2 (5.6)	11 (30.6)	7 (19.4)	8 (22.2)	8 (22.2)	37	0.285
No	128 (8.8)	242 (16.7)	313 (21.5)	364 (25.1)	406 (27.9)	1482	
Acne							
Yes	37 (7.0)	91 (17.3)	122 (23.2)	128 (24.3)	148 (28.1)	538	0.373
No	94 (9.7)	162 (16.8)	198 (20.5)	246 (25.5)	266 (27.5)	984	
Other health issues							
Yes	14 (10.1)	15 (10.9)	21 (15.2)	32 (23.2)	56 (40.6)	144	0.004
No	116 (8.6)	238 (17.6)	299 (22.1)	340 (25.2)	358 (26.5)	1375	
Diseases of the musculoskeletal system							
Yes	5 (4.6)	18 (16.7)	17 (15.7)	33 (30.6)	35 (32.4)	110	0.172
No	125 (9.0)	236 (17.1)	303 (21.9)	339 (24.5)	379 (27.4)	1410	

## Data Availability

Data are contained within the article.

## Appendix A

Questions from POLKA 18 questionnaire retrieved for the purpose of this study.

**1. How old are you?**
a. less than 18
b. 18
c. 19
d. over 19

**2. Gender:**
a. Female
b. Male
c. Other

**3. I live in:**
a. a city with more than 100,000 inhabitants
b. a city with less than 100,000 inhabitants
c. a rural area

**4. During your period have you ever:**
- missed physical education classes?—Yes/No - missed school activities other than physical education classes?—Yes/No

**6. How do you rate the painfulness during your periods? (scale 1–5)**
Not painful 1 2 3 4 5 very painful

**7. Have you ever smoked cigarettes?**
a. yes
b. no

**8. In the last 30 days, on how many days have you had a cigarette?**
a. 0
b. less than 1
c. 1
d. 2–5
e. 6–10
f. 11–20
g. more than 20

**9. In the last 30 days, on how many days did you drink at least one alcoholic drink?**
a. 0 days
b. 1–2 days
c. 3–5 days
d. 6–9 days
e. 10–19 days
f. 20–29 days
g. Daily

**10. Have you ever been drunk?**
a. yes
b. no

**11. Have you ever tried marijuana?**
a. yes
b. no

**12. Have you ever tried drugs other than marijuana (e.g., LSD, cocaine, ecstasy, hashish, heroin)?**
a. yes
b. no

**13. In the last year, have you experiences:**
- Panic attacks—Yes/No - Anxiety—Yes/No - Suicidal thoughts—Yes/No - Self-harm—Yes/No

**14. On a scale of 1 (very low)—10 (very high), how would you rate your stress level?**
very low 1 2 3 4 5 6 7 8 9 10 very high

**15. Have you ever been forced to do an activity you did not want to do—like kissing, touching or sexual intercourse?**
a. Yes
b. No

**16. Have you ever been forced to do an activity you did not want to do—like kissing, touching or sexual intercourse by someone you were in a relationship or a close relation with?**
a. Yes
b. No

**17. Have you ever experienced the following from someone you were in a relationship or close relation with:**
- **Physical violence**
a. Yes
b. No
- **Psychological violence**
a. Yes
b. No
